# Supplementary material for: Formation and retrieval of cell assemblies in a biologically realistic spiking neural network model of area CA3 in the mouse hippocampus
Source: J Comput Neurosci. 2024 Sep 17;52(4):303–21. doi: 10.1007/s10827-024-00881-3 (PMC11470887; doi:10.1007/s10827-024-00881-3)
Supplement: Supplementary file 1 — Supplementary Material 1 [file 10827_2024_881_MOESM1_ESM.docx]

**Supplementary Figure 1**


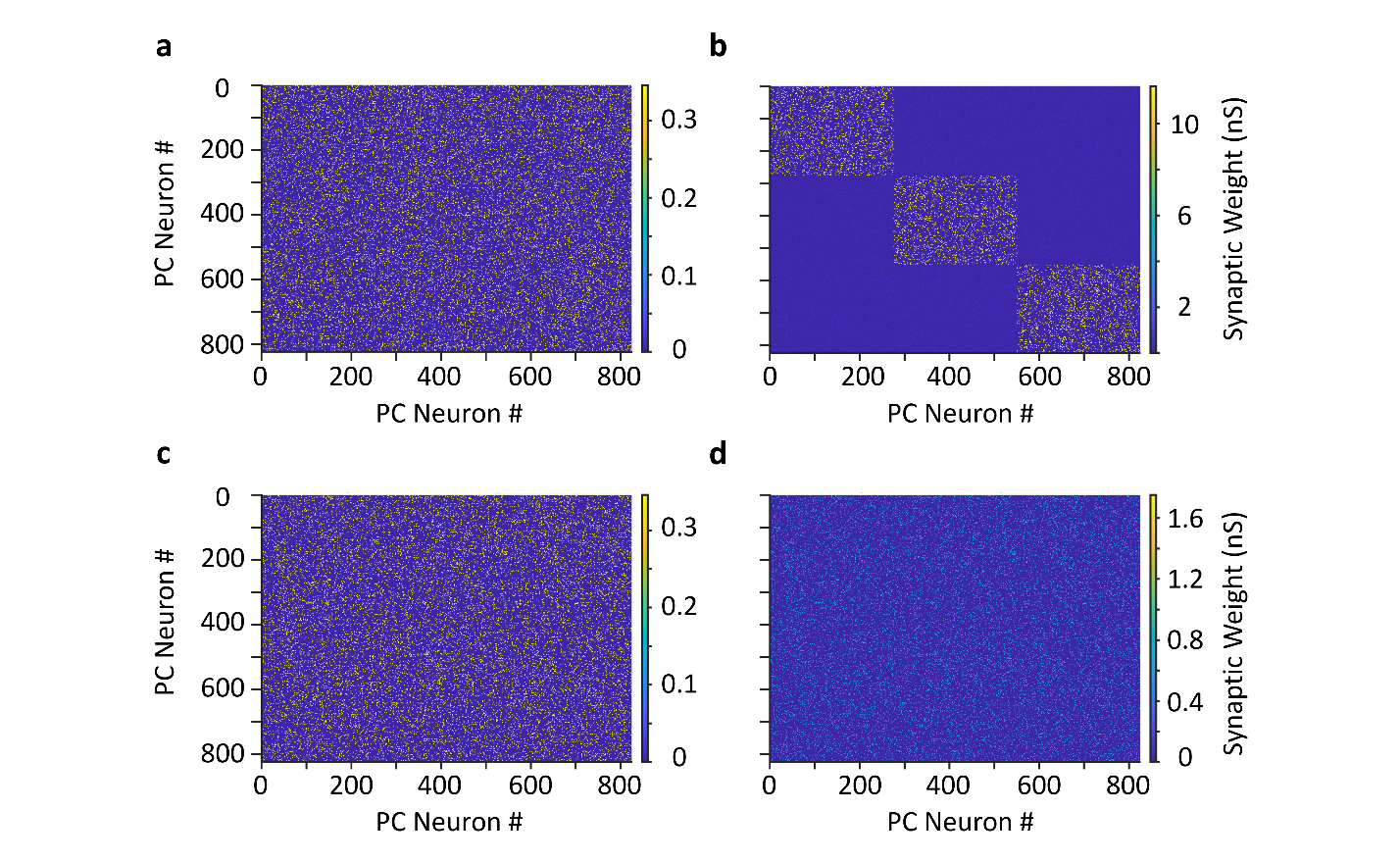


Supplementary Figure 1: Adjacency matrices graphically display auto-association in the CA3 SNN. (a,b,c,d) PC-PC synaptic weights between assembly (a,b) and non-assembly (c,d) members before (a,c) and after (b,d) training of the SNN with 65 repetitions of the patterns. Presentation of the pattern sets induced auto-association in the form of ‘blocks’ in the PC-PC synaptic weight matrix in (b).

**Supplementary Figure 2
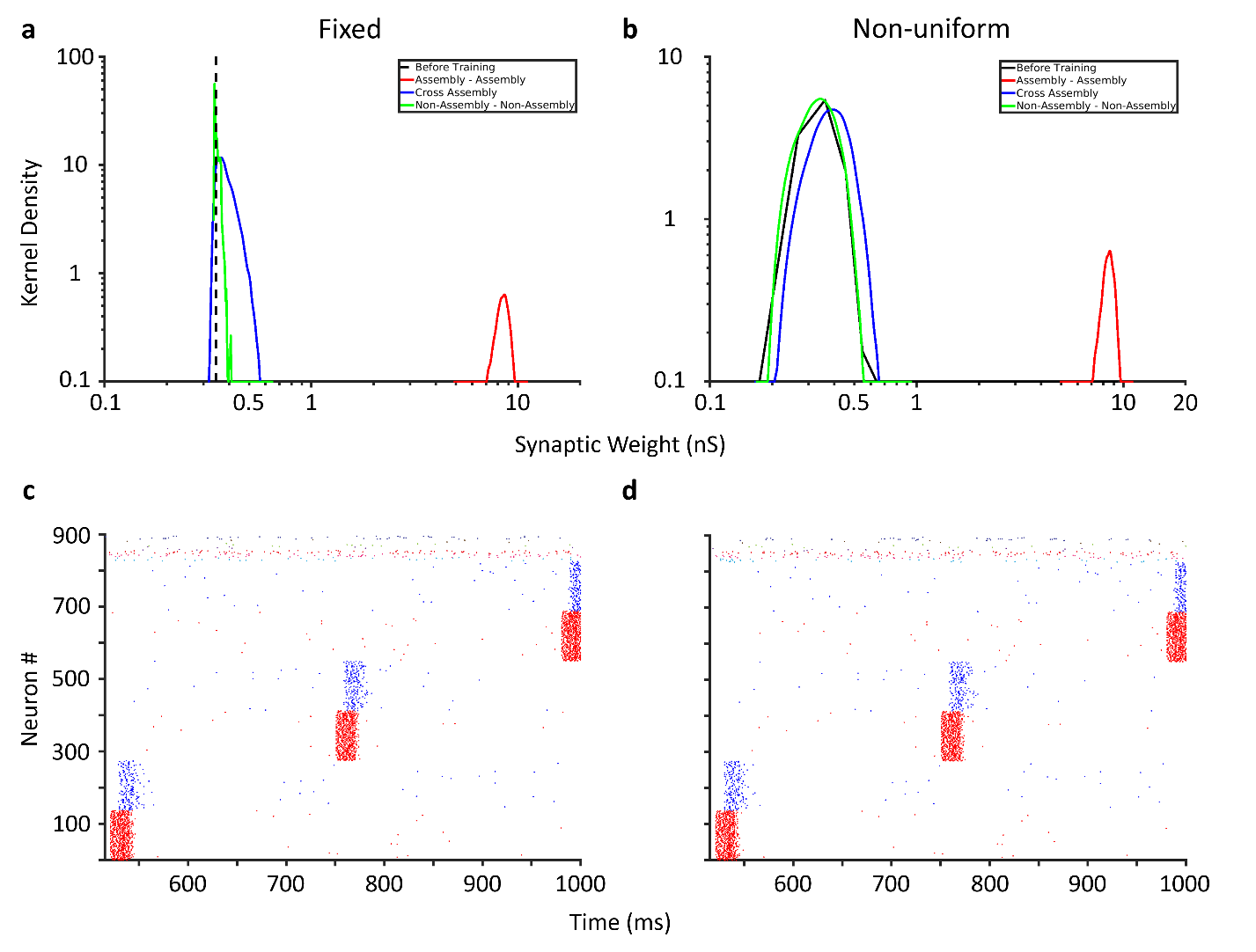
**

Supplementary Figure 2: Pattern completion is essentially unaltered when utilizing a normal (as opposed to constant) distribution of initial PC-PC weights. (a) Kernel density estimates of PC-PC synaptic weights between the 825 assembly member PCs (red), between assembly members of different assemblies (blue), between non-assembly members (green), and of synaptic weights from each group before training commenced (dashed black). (b) Kernel density estimates with a normal distribution before training commenced (solid black). (c,d) Activity from 825 Pyramidal cells (PC) and 10 interneurons of each interneuron type during the presentation of degraded input patterns. Input to 138 PCs (50% pattern degradation) in each assembly (red) leads to robust activation of the remaining assembly members (blue). Results in all panels are from an assembly size of 275 trained on 40 pattern presentations.

**Supplementary Figure 3**

**
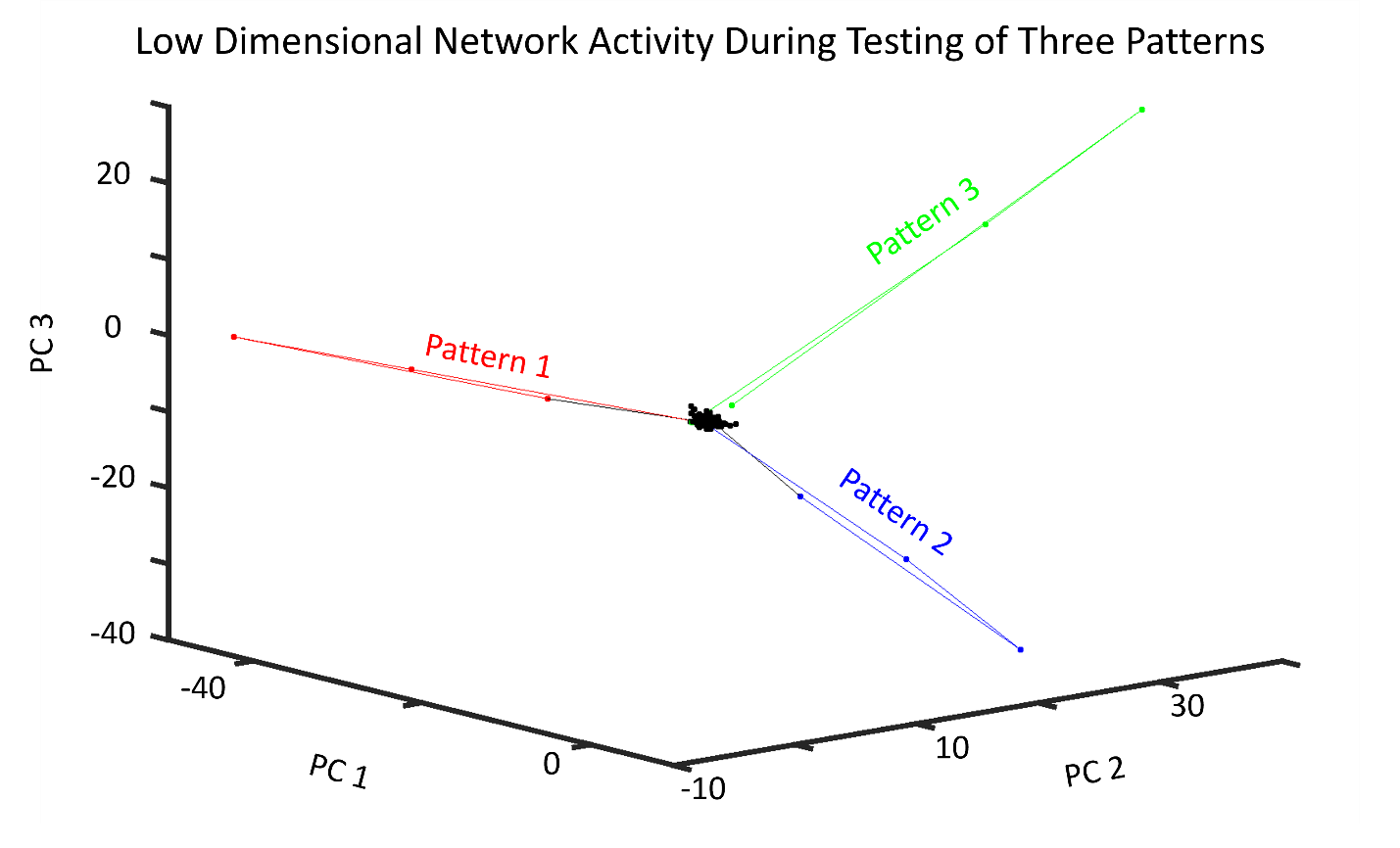
**

Supplementary Figure 3: Top 3 Principal Components of the network activity during one second of simulation testing pattern degradation for three patterns at 50% degradation with assembly size of 275 trained on 65 pattern presentations. The network converges towards a fixed-point attractor that retrieves each cell assembly in response to degraded forms of the first (red), second (blue), and third (green) input patterns.

**Supplementary Figure 4**


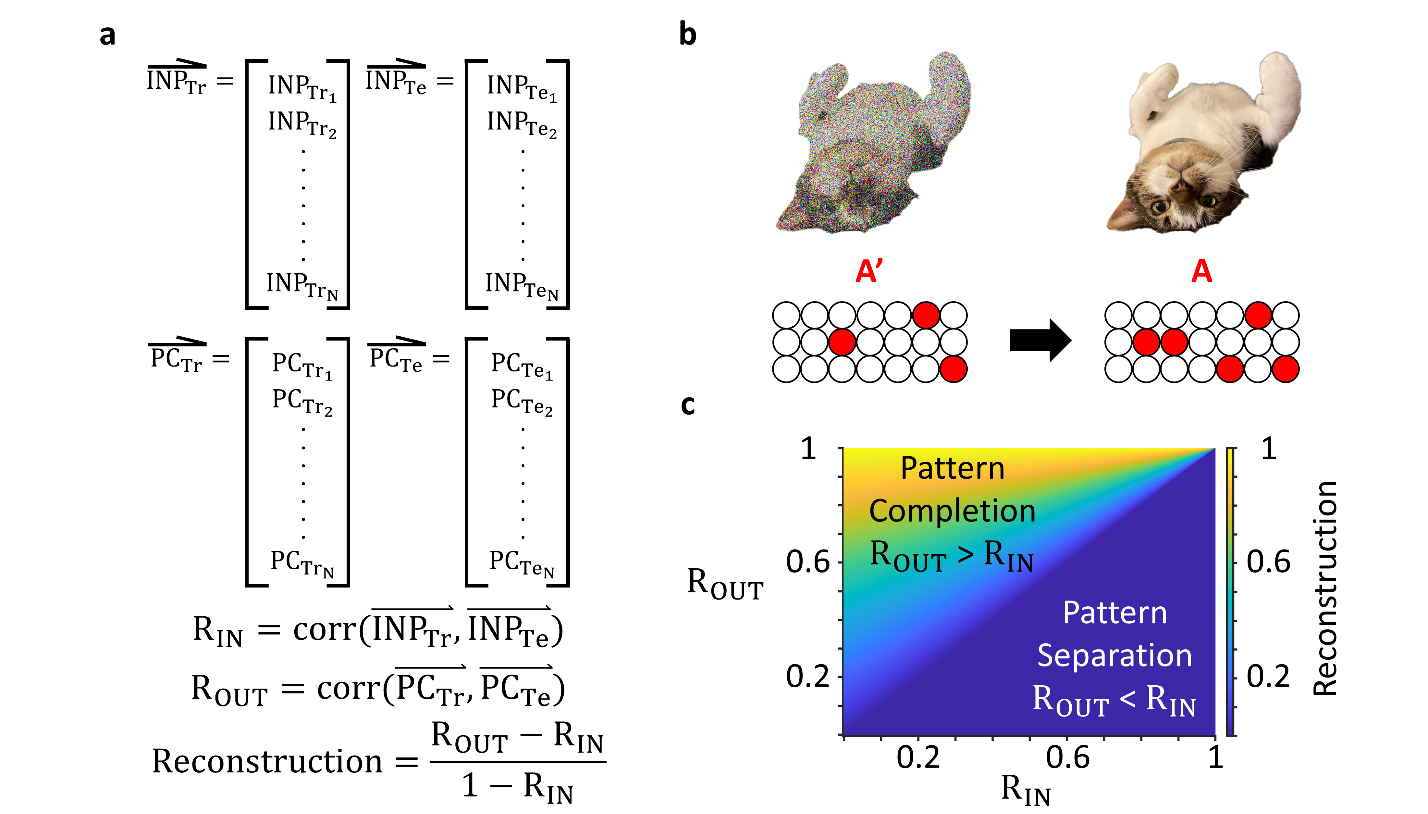


Supplementary Figure 4: Pattern reconstruction as a measure of pattern completion accuracy. (a) Pearson correlation coefficients (PCCs) are first computed from the training and testing input and training and testing output [45]. Pattern reconstruction is then calculated as the difference between output and input PCCs divided by the difference between the maximum PCC value (1) and the input PCC. (b) Schematic representation of two similar assemblies before and after pattern completion. A noisy input pattern (A’) is converted into a strong output pattern (A). (c) Analysis of pattern reconstruction based on input-output PCCs reveal regimes where pattern separation and pattern completion occur.

**Supplementary Figure 5**


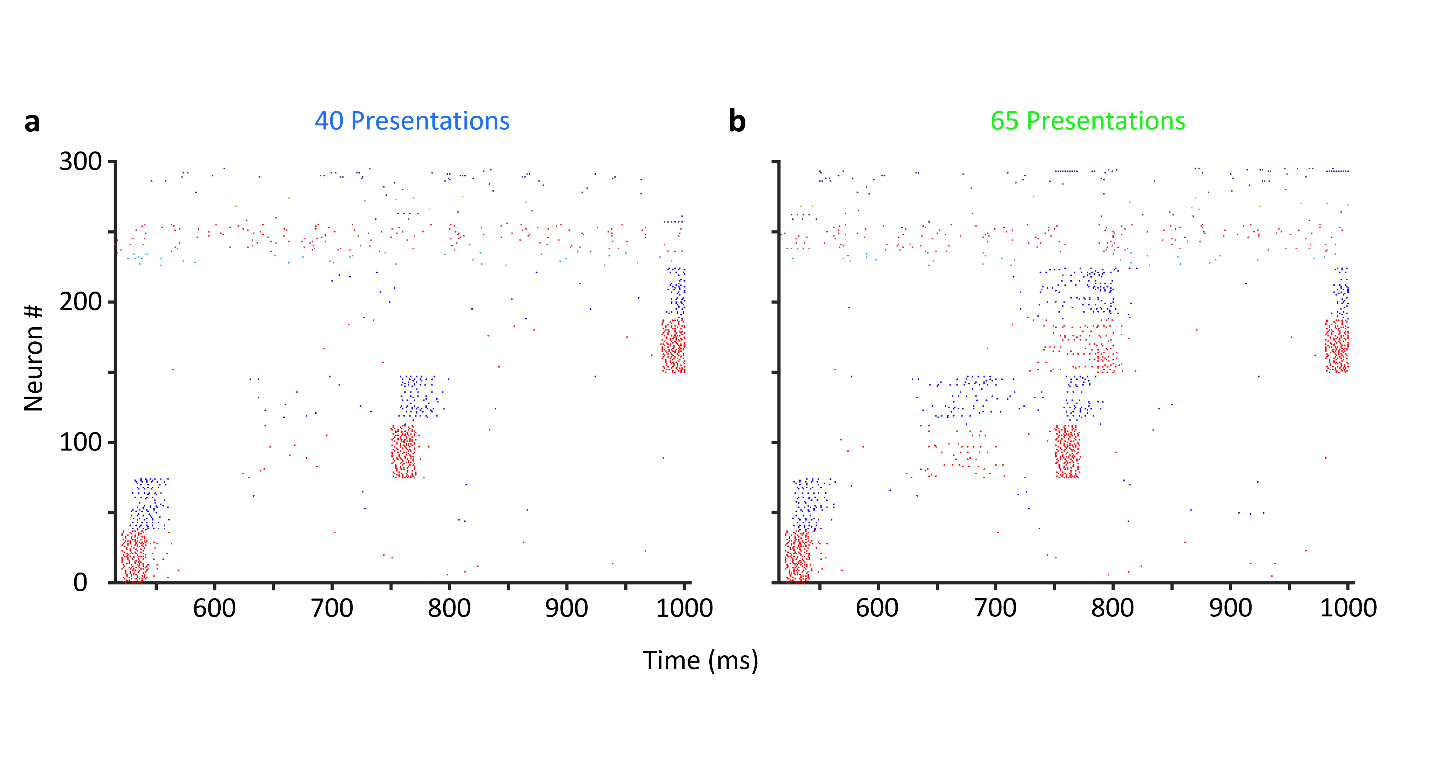


Supplementary Figure 5: Small assembly sizes exhibit pattern interference with increased training. Testing with pattern degradation of 50% and assembly sizes of 75 trained on (a) 40 and (b) 65 presentations.

**Supplementary Figure 6**

**
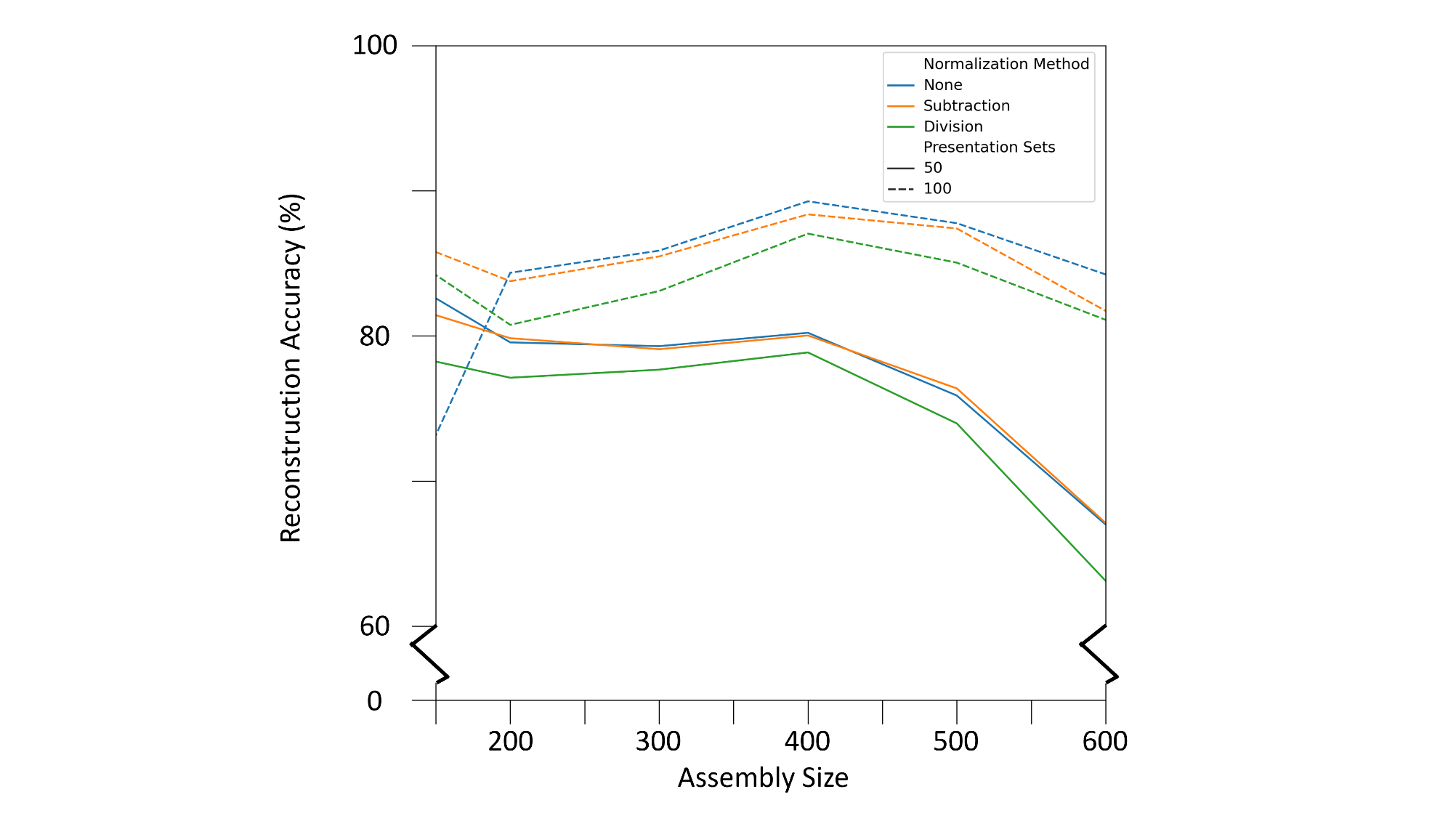
**

Supplementary Figure 6: Reconstruction accuracy with different downscaling methods to normalize the PC-PC synaptic weights throughout learning. The use of either subtractive or divisive normalization increases the range of assembly sizes that perform adequate pattern reconstruction (>= 50%) without pattern interference.
